# Low prevalence of organic pathology in a predominantly black population with premature adrenarche: need to stratify definitions and screening protocols

**DOI:** 10.1186/s13633-020-0075-8

**Published:** 2020-03-09

**Authors:** Christy Foster, Alicia Diaz-Thomas, Amit Lahoti

**Affiliations:** 1 Division of Endocrinology, Department of Pediatrics, University of Alabama at Birmingham, 1601 4th Avenue South, Birmingham, AL 35233 USA; 2 Division of Endocrinology, Department of Pediatrics, University of Tennessee Health Science Center, Memphis, TN USA

**Keywords:** Premature adrenarche, Black, Race, Non-classical CAH, NCCAH, Prevalence, Pathology, Screening

## Abstract

**Background:**

Premature adrenarche has been described as clinical and biochemical hyperandrogenism before the age of 8 years in girls and 9 years in boys and absence of signs of true puberty. Adrenal pathology such as adrenal tumors or non-classical congenital adrenal hyperplasia (NCCAH) and exogenous androgen exposure need to be excluded prior to diagnosing (idiopathic) premature adrenarche. Premature adrenarche is more common among black girls compared to white girls and other racial groups. Adrenal pathology such as NCCAH is less common as a cause for premature adrenarche compared with idiopathic premature adrenarche. The evaluation guidelines for premature adrenarche however are not individualized based on racial/ethnic differences. Few studies have been done to evaluate a largely black population with premature adrenarche to assess the incidence of adrenal pathology.

**Methods:**

This cross-sectional retrospective study evaluated characteristics of prepubertal patients seen in an endocrine clinic for premature adrenarche.

**Results:**

Two hundred and seventy three subjects had signs of early adrenarche. Three subjects were found to have CAH (2 with NCCAH and 1 with late diagnosis classical CAH). None were black. Exogenous androgen exposure was etiology in 4 additional subjects. These 7 patients were excluded from further analysis. The remaining subjects had idiopathic PA (*n* = 266); 76.7% were females. The mean age at initial visit was 6.42 +/− 1.97 years (with no racial difference) although black subjects were reported symptom onset at a significantly younger age compared to non-Hispanic white patients.

**Conclusions:**

Our study showed organic pathology was very uncommon in a predominantly black population with premature adrenarche. Patient factors that influence the probability of an underlying organic pathology including race/ ethnicity should be considered to individualize evaluation.

## Background

Adrenarche describes changes that occur in the adrenal cortex leading to increased production of adrenal androgen precursors in mid-childhood. Key hormones produced by the adrenal cortex include androgens such as testosterone as well as androgen precursors including dehydroepiandrosterone (DHEA), DHEA-sulfate (DHEAS), and androstenedione. Recent literature has also described 11-ketotestosterone as a key bioactive androgen in premature adrenarche [1, 2]. The clinical condition of premature adrenarche (PA) is defined as the appearance of clinical signs of androgen action before the age of 8 years in girls or 9 years in boys and absence of breast development in girls or genital enlargement in boys [3, 4]. PA is the result of early activation of the zona reticularis in the adrenal gland resulting in a rise of androgens [5]. To make the definitive diagnosis of PA, precocious puberty, exogenous androgen exposure, adrenocorticoid and gonadal sex hormone secreting tumors and non-classic congenital adrenal hyperplasia (NCCAH) must be excluded as these conditions can also result in clinical signs of early adrenarche [6].

The incidence of PA has been shown to be significantly higher in girls than boys [7, 8] and may also be mediated by a higher BMI [9]. In a study by *Rosenfeld* et al, the prevalence of adrenarche (defined as pubic hair stage 3 in the study) was < 1% in 8-year-old girls with normal BMI (all ethnicities taken together). However, among these healthy weight 8-year-old girls, non-Hispanic black girls had the highest prevalence of 3% and non-Hispanic white (NHW) girls had the lowest prevalence of 0.01%. The prevalence was higher at 2% in 8 year old girls (all ethnicities taken together) with a BMI >85th percentile. In 10 year old boys with a normal BMI, adrenarche occurred in ~ 1% (all ethnicities taken together) but increased to 2.4% in boys with BMI ≥ 85th percentile [9]. Another large study had suggested a higher prevalence of PA in girls of African-American origin compared to NHW girls [10].

Limited data is available regarding the incidence of adrenal pathology presenting as PA. The incidence of NCCAH in the setting of PA has been reported to be about 5% in prior studies [11]. Prior studies have shown the prevalence of NCCAH was 4–10% in Mediterranean populations [12–14]. In the Turkish population, NCCAH was identified in 4% of patients diagnosed with either precocious adrenarche, PCOS or hirsutism [15]. In another study of 28 Brazilian girls, 6 girls were diagnosed with NCCAH based on stimulated 17-OH progesterone (17-OHP) levels [16]. In black patients with PA however, the incidence of adrenal pathology is inadequately studied.

Evaluation for other adrenal pathology is key, given that PA is a diagnosis of exclusion. In patients with idiopathic PA, DHEAS concentrations are in the mid or late pubertal range [17]. Based on one study, a DHEAS level of > 40 μg/dL (LC-MS) is considered adrenarchal [3, 18, 19]. Basal levels of 17-OHP > 2 ng/mL (200 ng/dL) has been identified as a specific (93%) and sensitive test (100%) for NCCAH [20]. Screening tests often done to evaluate PA include early morning measurements of DHEAS, androstenedione, 17-OHP, and testosterone in patients with PA [21]. A radiological assessment with a bone age is also sometimes utilized to assess exposure to androgens. However, an advanced bone age in PA is common, and even a bone age that is advanced by more than 2 years in a child without other worrisome findings is generally consistent with PA, with other adrenal pathology rarely found [22].

Studies performed by Pediatric Research in Office Settings Network found that the mean age of pubarche in black girls and boys is 1 year or more earlier than white children [10, 23]. It is unclear at this time, if the earlier onset of PA in black children is the new normal for this racial group or these patients are at risk of metabolic consequences and hyperandrogenic disorders associated with PA. In the absence of conclusive evidence, the age cut-offs for defining PA has not been modified based on ethnicity/ race.

Studies that describe PA in black patients are limited, and studies evaluating the incidence of NCCAH, adrenal or gonadal tumors or exogenous androgen exposure in patients evaluated for PA are even more rare. Our study aims to evaluate the incidence of PA and the incidence of diagnosis of other endocrine pathology in a racially mixed but predominantly black cohort of patients with PA.

## Methods

### Subjects and design

#### Study population and design

A chart review was performed for patients with PA (adrenarche before the age of 8 years in girls and before the age of 9 years in boys). Subjects had been evaluated at the Le Bonheur Children’s Hospital between January 1st 2015 and January 31st 2018. Potential subjects were identified using ICD-9 codes: 259.1, 255.2, 255.8 and ICD-10 codes: E30.8, E30.1, E25.0, E27.8. Four hundred and ninety patients meeting these ICD codes were identified and 273 were diagnosed with early signs of adrenarche. Race was self-reported as non-Hispanic white (NHW), black and other. The institutional review board approved the retrospective review of patients’ clinical charts and informed consent was not required.

Anthropometric data including BMI, height, weight and blood pressure were extracted from the patient’s initial endocrine visit. Blood pressure measurements were taken with the patient in the sitting position. Elevated systolic blood pressure (SBP) or diastolic blood pressure (DBP) were defined as a value above the 95th percentile for age, gender and height percentile [24]. Clinical data included signs of androgen action, including apocrine body odor, acne, and pubic or axillary hair. Central puberty was defined as breast development at Tanner 2 or greater for girls and testicular volume >/= 4 cc for boys using a Prader orchidometer.

Testosterone, 17-OHP, DHEAS and androstenedione were measured by tandem mass spectroscopy at *Esoterix*® laboratories.

### Statistical analyses

Statistical analyses were carried out using SPSS statistical version 25 software. Data are expressed as mean +/− standard deviation. BMI values were converted into standard deviations scores (SDS) that were normalized for age and gender based on 2000 Centers for Disease Control (CDC) growth charts. Differences between gender for the patients with PA were compared using unpaired student t-tests. Differences between the ethnic groups were compared by one-way analysis of variance; Boneferroni’s post-hoc test was applied when appropriate.

## Results

Our study identified 273 subjects with symptoms of early adrenarcheThere were 266 patients with the diagnosis of idiopathic PA after these patients were excluded. These patients were excluded from further analysis. Of the subjects with idiopathic PA (*n* = 266), 76.7% female were female and the mean age of their initial visit was 6.42 +/− 1.97 years. The mean systolic blood pressure was 103 +/− 8 with a mean BMI z-score of 1.24. The incidence of hypertension was 11.9%. In our cohort, we identified 3 subjects with late-onset congenital adrenal hyperplasia. One patient was a NHW female who presented with anal hair and body odor at age 6. Newborn screen was normal. Her unstimulated morning 17-OHP at initial evaluation was elevated to 1260 ng/dL (ref: < 91 ng/dL). On her stimulatory testing, her 17-OHP rose from 659 to 6250 ng/dL. Her cortisol rose from 5.8 mcg/dL to 12 mcg/dL. She was diagnosed with non-classic 21-hydroxylase deficiency and hydrocortisone therapy was initiated. The second subject was a previously healthy, NHW male with symptoms of apocrine body odor, pubic hair and penile erection at 2 years age. Additionally, he had rapid growth and excessive weight gain (five inches of height and twenty-two-pound weight gain in a year). Given family history of a maternal cousin with classical CAH, an ACTH stimulation test was performed which showed 17-OHP rise from 3580 ng/dL to 22,300 ng/dL. Serum cortisol rose from 6 μg/dL to 8.1 μg/dL. He had a markedly advanced bone age > 2 SD and an elevated androstenedione. He was diagnosed with non-classical CAH and started on hydrocortisone therapy. Genetic testing showed two alterations in the 21-hydroxylase gene. The third subject was a Hispanic White female born in Mexico and was evaluated at 25 days of life for clitoromegaly with a 17-OHP of 77 ng/dL (ref: 0.06–40.41). No history of electrolyte abnormalities. She continued to have clitoral enlargement and pubic hair growth began at 4.5 years age. Upon initial endocrine evaluation in US, at 5.5 years, her baseline 17-OHP level was 14,000 ng/dL (< 91 ng/dL). Her total testosterone was 113 ng/dL and cortisol level was 6.1 mcg/dL. ACTH stimulation test was not performed. She was classified as classical CAH (late diagnosis) and was started on hydrocortisone and fludrocortisone replacement. The age of presentation, age of described initial symptoms, blood pressure, weight SDS, height SDS, BMI SDS were not significantly different for these subjects than the rest of the cohort described below. There were expected differences between testosterone, 17-OHP, and androstenedione levels between the three patients with adrenal pathology and the rest of the cohort. These 3 patients along with 4 others with exogenous androgen exposure were excluded from further analysis.

The remaining patients (*n* = 266) were diagnosed with idiopathic PA and are described further in the sections below. Patients were predominantly females (76.7%) and the mean age at their initial visit was 6.42 +/− 1.97 years. The mean age of initial symptoms was 4.7 +/− 2 years. The mean systolic blood pressure was 103 +/− 8 mmHg with a mean BMI z-score of 1.24 +/− 1.21. The incidence of hypertension was 11.9%. Table 1 summarizes the anthropometric data and hormone levels.

**Table 1:** Demographics of the patients evaluated for premature adrenarche (*n* = 266)

Characteristics	*n* = 266
Female % (n)	76.7 (204)
Race, % (n)	
Non-Hispanic white	20.7 (55)
Black	77.1 (205)
Hispanic white	2.3 (6)
Age at initial endocrine evaluation (years)	6.42 +/− 1.97
Age at initial symptoms (years)	4.7 +/− 2
Weight z-score	1.25 +/− 1.33
Height z-score	0.64 +/− 1.24
BMI z-score	1.24 +/− 1.21
Hypertension	11.6%
Tanner stage (pubic hair)	2.27 +/− 0.58

Values listed are means and standard deviation

Table 2 shows analysis of the differences of presentation of PA based on gender. There were 62 males and 204 females. Both groups had a similar racial distribution (data not shown). Age, Weight SDS, BMI SDS, and tanner staging were not significantly different between the two genders. Males had a significant higher level of DHEAS, which would be expected given that DHEAS is higher in males than females at each Tanner stage. There were no differences in testosterone, 17-OHP, and androstenedione levels between the two groups.

**Table 2:** Comparison of clinical characteristics of patients with premature adrenarche based on gender

	Male (*n* = 62)	Female (*n* = 204)	
Age at initial evaluation (years)	6.69 +/− 2.37	6.33 +/− 1.83	NS
Age at initial symptoms (years)	5.08 +/− 2.41	4.58 +/− 1.89	NS
Weight z-score	1.40 +/− 1.39	1.21 +/− 1.32	NS
BMI z-score	1.38 +/− 1.27	1.21 +/− 1.20	NS
Systolic BP	103 +/− 7.4	103 +/− 8.03	NS
Diastolic BP	65 +/− 6.7	64.5 +/− 6.70	NS
Hypertension	12.9%	12.3%	NS
Tanner stage (pubic hair)	2.18 +/− 0.61	2.31 +/− 0.57	NS
Testosterone (ng/dL)	7.64 +/− 5.56	7.68 +/− 10.0	NS
17-OH progesterone (ng/dL)	27.91 +/− 36.34	28.65 +/− 22.87	NS
DHEA-sulfate (ug/dL)	109.8 +/− 63.4	72.42 +/− 52.76	< 0.001*
Androstenedione (ng/dL)	20.29 +/− 7.27	18.89 +/− 8.59	NS

Values listed are means and standard deviation

*NS* not significant

*Statistically significant difference at the *P* < 0.05

Racial differences among the NHW, black and other groups in the cohort are presented in Table 3. There were no significant differences between age of presentation, percentage females, weight SDS, and BMI SDS. Black patients were found to describe symptoms at a significantly younger age than NHW patients. Systolic blood pressures were significantly higher in NHW patients compared to black patients. Testosterone, DHEAS, 17-OHP and androstenedione levels were not significantly different between populations. Looking specifically within the male population, there was no significant difference in hormone levels between races. Interesting, in the female population, NHW females had significantly higher DHEAS levels than black females.

**Table 3:** Comparison of clinical characteristics of patients with premature adrenarche based on race

	non-Hispanic white (*n* = 55)	Black (*n* = 205)	Other (n = 6)	*p*-value
Female % (n)	80 (44)	75.6 (155)	83.3 (5)	NS
Age at initial evaluation (years)	6.67 +/− 1.83	6.30+/− 2.02	7.94 +/− 1.00	NS
Age at initial symptoms/ signs (years)	5.42 +/− 1.89	4.44 +/− 2.01	6.48 +/− 2.15	0.004*
Weight z-score	1.32 +/− 1.07	1.25 +/− 1.39	0.84+/− 1.56	NS
BMI z-score	1.28 +/− 0.92	1.24 +/−1.29	1.33 +/− 0.83	NS
Systolic BP	106 +/− 7.44	103+/− 7.85	101.29 +/− 8.83	0.008*
Diastolic BP	65.5 +/− 6.47	64.4 +/−6.77	67.0+/− 6.03	NS
Hypertension % (n)	16.4 (9)	11.2 (23)	16.7 (1)	NS
Tanner stage (pubic hair)	2.09 +/− 0.53	2.33 +/− 0.59	1.83 +/− 0.41	0.006*
Testosterone (ng/dL)	11.2 +/− 17.0	6.78 +/− 5.32	6.97 +/− 1.50	NS
17-OH progesterone (ng/dL)	34.0 +/− 35.6	26.4 +/− 24.1	35.0 +/− 9.64	NS
DHEA-sulfate (ug/dL)	97.7 +/− 78.7	76.0+/− 48.6	108 +/− 102	NS
Androstenedione (ng/dL)	18.0 +/− 9.46	19.5+/− 8.05	20.5 +/−9.19	NS

Values listed are means and standard deviation

*NS* not significant

*Statistically significant difference at the *P* < 0.05

## Discussion

The present study demonstrates that organic pathology has a low incidence in a primarily black population with PA; less than 1% of subjects that were found to have NCCAH/ late diagnosis CAH, none of which were black. These findings concur with prior studies describing an overall low incidence of NCCAH in PA patients [25]. Based on our study, there does not appear to be a significant difference in the age of presentation or onset of symptoms in patients identified to have NCCAH compared to those with idiopathic PA, however comparison was difficult due to the very small number of patients with NCCAH in the cohort. Screening androgens were found to be significantly higher in subjects with NCCAH compared to those patients with PA. These findings are similar to what was found in a 2012 study by von Oettingen et al. [26]. Given our low incidence of adrenal pathology, it was difficult to draw conclusions on clinical characteristics which may help distinguish those with NCCAH from those with idiopathic PA. None of the patients were diagnosed with a virilizing tumor.

Our study also shows that females with PA had symptom onset and clinical presentation at a significantly younger age compared to males. Some of this difference is expected due to the gender difference in the age cut-offs used for definition of PA. Our study showed that males with PA have significantly higher DHEA-sulfate levels compared to females. Prior studies have shown similar findings [26]. This sexual dimorphism of adrenarche might be secondary to gender differences in peripheral androgen metabolism.

Our study also examines racial differences in PA. No black subjects in our study with PA were found to have adrenal, gonadal or hypothalamic/pituitary pathology. Newborn screening data for CAH shows an incidence of 1:24,840 in black newborns screened from 2007 to 2014, which is lower than in NHW and Hispanic white populations [27]. Our study shows a low prevalence of NCCAH in black population with PA as well. While, prior studies looking at racial differences are limited, our cohort reinforces the findings of a low prevalence of pathological causes of PA in another study that evaluated black subjects [28, 29]. *Herman-Giddens* et al showed that 1% of black females and only 0.26% of NHW girls had pubic hair at 3 years of age. The mean age for stage 2 pubic hair development was 8.78 years for black females, and 10.51 years for NHW females respectively [10]. The mean age for development of stage 2 pubic hair is 10.2 years for black males and 11.4 years for NHW males [23]. Hence while it is recognized that black subjects have earlier adrenarche and have a low prevalence of other adrenal pathology like NCCAH, currently accepted definitions for what is considered premature do not take race into account. These findings along with the findings in our study suggest the need to consider revising the definition of PA and the paucity of data in this field highlights the need of more studies. Furthermore, black subjects described symptoms earlier but did not seek care any early than their NHW counterparts. Evaluation of this might be an interesting question that would merit more study.

Excessive testing can lead to unnecessary cost. Preliminary cost analysis of screening for PA showed there is a significant cost per patient despite wide variation in costs. From personal communications with a major laboratory in United States (as of May-Sep 2019), the estimated price charged for serum testosterone was 150 USD, 121.75 USD for serum 17OHP 162 USD for serum androstenedione, and 110.25 USD for DHEAS (LC-MS/MS methodology), total 544 USD. Prices charged by another major laboratory in United States were 220.47 USD for serum testosterone (LC-MS/MS), 199.10 USD for serum 17OHP (LC-MS/MS), 253.09 USD for androstenedione (LC-MS/MS) and 213.72 USD for DHEAS (immunoassay), total 886.38 USD. Each cortisol level is 113 USD. Total price for these labs in self-pay patients at our institution was approximately 478 USD. Using the CPT code 82533, the charge for this testing was 5.187.80 USD. In addition to these costs, there are additional costs associated with the costs of an endocrine consultation, charges of phlebotomy, wage lost for parents coming for consultation or testing. A true estimate of the magnitude of these costs would require multiplication with the number of patients evaluated and a detailed analysis, which is beyond the scope of this paper.

Our study has several advantages. This is one of the few studies on PA in a primarily black population and thus adds to the body of literature for this racial group. Since all patients are from a single institution, almost all laboratory tests were done at a single hospital contracted reference laboratory with LC-MS/MS methodology, minimizing assay variations in test results. There are also some limitations to our study. First, the study involves only one institution and therefore all of the findings may not be generalizable. Secondly, there is some heterogeneity in the practice of evaluating PA within our practitioners and not all patients had labs obtained on their initial evaluation. Laboratory blood draws were not uniformly performed at 8 am, possibly impacting androgen levels. Third, our study has a small sample size and may make identifying racial differences more challenging. Finally, our cross-sectional design does not allow for the evaluation of the natural history or evolution of the condition.

## Conclusions

In conclusion, idiopathic PA is a diagnosis of exclusion. Our study suggests that organic pathologies are uncommon among black patients with PA. Since adrenarche is noted to occur earlier in black girls and boys, they also represent the larger proportion of patients evaluated for PA. NCCAH, the most common pathology being screened, is overall rare in this racial group. A multi-center, prospective, longitudinal study with standardized 8 am laboratory evaluation in a predominantly black population with PA would allow us to further understand disease frequencies and any gender differences in the natural history of PA. PA evaluation guidelines based on these additional studies could help determine which patients require immediate laboratory investigations and which could be managed by conservative serial exams by the primary care provider.

## Data Availability

The datasets used during and/or analyzed during the current study are available from the corresponding author on reasonable request.

## Abbreviations

17-OHP: 17-hydroxy progesterone
BMI: Body mass index
DBP: Diastolic blood pressure
DHEA: Dehydroepiandrosterone
DHEAS: DHEA-sulfate
NCCAH: Non-classical CAH
NHW: Non-Hispanic white
PA: Premature adrenarche
SBP: Systolic blood pressure
